# Annotations

**Published:** 1899-02-11

**Authors:** 


### Annotations.

### The Progress of the Crusade against Consumption.

The main facts about tuberculosis and the manner in which its infection is disseminated are being well rubbed into the public mind. In the pulpit, in the daily press, by lectures, and by magazine articles, the crusade against tuberculosis is being preached on every side, and, perhaps, here and there the preaching falls upon good ground and brings forth personal endeavour to combat the disease. We fear, however, that, so far, the tendency has been rather in the direction of urging each other to do something than of doing it ourselves, of demanding some great scheme rather than of each doing what we can to protect ourselves and our families from the infection. A lecture given at the Society of Arts by Mr. Hunting a week or two ago gave an admirable demonstration of the extent to which infection is distributed by milk. He pointed out that at least 20 per cent. of the cattle over two years old were more or less affected with tuberculosis, and that an enormous stream of infectious milk was being poured into our cities.

He is not the first, however, who has told us these things. They are now well known—so well known, in fact, that people have come in a theoretical kind of way to recognise that "something must be done"; and, indeed, the very object of the lecture was to show what that something was, and what it would cost to exercise some sort of check by slaughter on the progress of tuberculosis among cattle, as is done in regard to some other infectious diseases. About £100,000 the first year, and probably rather less in years to come, is the estimate for compensation for compulsory slaughter of infected animals, and it is quite possible that by dint of much preaching some such course will sooner or later be adopted. It is just the sort of thing that people who do not want to be bothered are ready to do. Pass an Act, put the responsibility on some official, and get rid of the subject. That is not the way, however, in which tuberculosis will be suppressed. The whole people must co-operate if successful war is to be waged; and it must strike the observer as curious, and not altogether encouraging, to note how little headway the "crusade" has so far made in ordinary matters of dai y life. How many more people boil their milk now than did so a few months ago? Yet the very Commission which told us about the serious danger attaching to the use of milk told us almost in the same breath that boiling it even for an instant would entirely prevent the transmission of infection by its means.

Do we see any less spitting in streets or omnibuses than we did? How many times a week, a month, or even how many times at all has one seen a pocket spittoon used in a public place by any member of the immense army of consumptives who we know are wandering about in our midst? Do we see any signs of fashion issuing an edict against fluffy carpets and soft hangings? Do we see mistresses insisting on their housemaids using a damp cloth instead of a duster? Do we see any fewer knicknacks in drawing-rooms, or any more bed-rooms with painted walls and linoleum floors? Do we see our places of public resort, our railway carriages, and especially our theatres upholstered in washable material? Lastly, even in our hospitals and infirmaries, how often do we find the managers taking care that the meat supplied to the patients comes from non-tuberculous animals, and that the milk is derived from cows whose freedom from tubercle has been proved by the tuberculin test? Nay, we will go further, and ask in how many workhouse infirmaries—the last resort of so many consumptives—is any effort made to separate these patients from those suffering from other classes of disease, and in how many asylums, whose inmates notoriously suffer largely from consumption, are measures taken to isolate those who are affected, and who, from their mental condition, cannot be taught to adopt proper measures to lessen the danger to which their fellow-inmates are exposed? Here and there a little is being done, but we doubt whether the "crusade" has yet to any large extent touched the heart of the people or even of those who occupy responsible positions. On every hand the doctrine of infectivity is being accepted as "most interesting" and as showing clearly what somebody else should do. On every hand do we see signs of an increasing desire for some great thing being done preferably by "the Government" or "the authorities," but nowhere do we see any marked willingness to incorporate the doctrine of the infectivity of tuberculosis as a guiding principle in the doings of our daily life, and that is what is wanted if the "crusade" is to come to full fruition.

### Voluntary Effort and State Control.

The good work which is done by voluntary effort, even in regard to charities which are under State control, is well illustrated by the attempts now being made by the State Charities Aid Association of New York to stem the frightful mortality which has prevailed among the motherless infants cared for by that city in the Infants' Hospital on Randall's Island. It appears that among these foundlings the death-rate had been appalling. During the year ending September 30th, 1895, there were received 129 foundlings, of whom four were reclaimed almost immediately by their parents, one was adopted the day after its admission, and the other 124 died—a death-rate among those who did not leave the institution almost immediately after admission of 100 per cent. The next year 131 foundlings were received, of whom six were adopted, one lived to be two years old and was transferred to another institution, and the other 124 died—a death-rate among those not adopted of 99·2 per cent. Again nearly the same tale

ANNOTATIONS—*continued.*

had to be told of 1897. Practically all the foundlings who were not adopted or reclaimed by their parents died. This was not to be tolerated, for it was known that other large cities, notably Boston and Philadelphia, finding the death-rate among similar children in their public institutions equally high, had tried the plan of boarding the foundlings in families, and had reduced their mortality from 95 and 100 per cent. to between 20 and 30 per cent. A committee was therefore appointed, and, after some search, a number of families were found among whom to board out the infants. The services of an experienced agent were obtained to investigate the surroundings, and to personally inspect the homes in which the children were to be placed. Forty-five infants were received from the Infants' Hospital and placed with these foster parents, many of them being at the time of their removal in a very bad, and some in an apparently hopeless condition. They have been under the constant supervision of the agent of the committee and under the care of the best physicians in their localities. It is yet too early to say what degree of success will attend the movement, but of the 45 children received 16 were still living at the end of September. That, however, is not all the good that has been done by the matter being taken up by the Association, for the attention of the hospital authorities has been so strongly drawn to the terrible mortality that was going on, that a marked improvement has already been made in the institution itself, especially in the direction of providing the infants with wet nurses. The action of the Association is, however, but another example of the great advantage of outside voluntary agencies taking an interest in the working of all State-supported institutions.

### A "White List."

While many social workers find out in the course of their investigations that certain firms—some with a good reputation, some with a bad one—treat their workers harshly and unjustly, they are restrained by a fear of the law of libel from giving the publicity they would desire to the names of these. When a firm's bad treatment of its employés leads it into the police-court, for neglect of sanitary precautions, keeping women and young persons at work for hours longer than are legally permissible, or the like, it may be safe to speak, but not otherwise. Therefore, it is impossible, or at least injudicious, to publish a "black list" where the names of the guilty firms may be held up to reprobation. But there is nothing to hinder one from compiling and publishing a "white list," giving the names of those firms which, in any branch of industry, treat their workers fairly—*i.e.*, do not try to evade the law as to the number of hours worked by allowing workers to take home work after shop hours are over, keep their work-rooms in a comfortable and sanitary condition, nor otherwise take advantage of the poor creatures, "whose poverty but not their will consents" to put them at their mercy. This has been done by the Christian Social Union in Glasgow; and, though the compilation of the list involved a good deal of trouble, the results of doing so have been satisfactory, not only to those who for selfish or unselfish reasons have an objection to using sweated goods, but to the firms who have had the good fortune to find a place in the list. There is nothing libellous in the process. You remark that Codlin is the friend, but you never hint that Short is not. Short may be the best of men, and nobody says that he is not—at least in public. No one says that he is hard upon his workers; but then nobody says he is the reverse. He is not black-listed. Only he is not on the white list.

### Medical Advertising.

There is much that is delightful in practising in foreign watering-places, and, indeed, to follow the weather, to live always where it is best and brightest, and still to have one's work at hand, and all the time, even while living among the idlers, to be occupied in one's profession, must seem to many a hardly-driven practitioner, who has to make the best of his English surroundings, to be an ideal way of earning a living, Much, however, would have more—that is proverbial; and it seems that some of these gentlemen whose lines have fallen in such pleasant places allow their lot to be rendered still more delightful by the fees attracted by the gentle art of advertising. We call it a gentle art because, like angling, which has always been so described, it is an endeavour to catch the unwary. Nay, these advertisers remind us of anglers in another way. Everyone knows that the latter maintain that it is quite a mistake to think the tortured worm is hurt when put upon the hook, and with the same sort of mental incapacity to see things as they are, we believe that these gentlemen are ready to maintain that their advertisements are not advertisements at all—only announcements. Still, like the little worms which to the common eye seem most uncomfortable, however much we may be told they are all right, so the list of members of the Continental Anglo-American Medical Society, as it appears week by week in the *English and American Gazette*, looks wondrous like an unblushing advertisement, however much those whose names appear may say they do not feel it in that light. At any rate, here is the fact that a weekly journal published in Paris, and "representing Anglo-Saxon interests on the Continent of Europe," publishes on its advertisement page a list of the members of the Continental Anglo-American Medical Society, the names being sorted out under the headings of the towns in which they practice. In this list appear the names of practitioners of various degrees, a good many of them being Fellows of the Royal College of Physicians of London. The advertising quality of the announcement is apparent at once when we mention that the list does not contain all those practising with English qualifications in the various towns; indeed, in regard to one well-known health resort in which, according to "Churchill's Medical Directory," there are four practitioners with English qualifications, only one name is mentioned in this advertisement, and that one a Fellow of the College of Physicians. No doubt when on pleasure bent many people do things abroad that they would not do in their own town. But these gentlemen are not on pleasure bent; they are seriously engaged in their professional work, and we cannot but see how great must be the difficulty of maintaining in the profession a proper feeling in regard to medical ethics while those possessing the highest qualifications are allowed, without any protest, to permit their names to be advertised in such a list.

